# Effect of EMG-activated vibrotactile biofeedback on skill learning in children with genetic and acquired dystonia during practice of a point-to-point and a cyclic task

**DOI:** 10.21203/rs.3.rs-6228869/v1

**Published:** 2025-05-07

**Authors:** Maral Kasiri, Emilia Ambrosini, Emilia Biffi, Shinichi Amano, Alessandra Pedrocchi, Nardo Nardocci, Elena Beretta, Giovanna Zorzi, Terence D. Sanger

**Affiliations:** University of California, Irvine; Politecnico di Milano; Scientific Institute for Research, Hospitalization and Healthcare (IRCCS) Eugenio Medea; University of Southern California; Politecnico di Milano; Istituto Neurologico Carlo Besta; Scientific Institute for Research, Hospitalization and Healthcare (IRCCS) Eugenio Medea; Istituto Neurologico Carlo Besta; University of California, Irvine

**Keywords:** Dystonia, Motor Control, Rehabilitation, Sensory Deficit, Vibrotactile Biofeedback

## Abstract

**Objective:**

Dystonia is a movement disorder causing involuntary muscle contractions and abnormal movements. Often, repeated practice does not lead to motor improvement in children with acquired dystonia, likely due to sensory deficits, which may contribute to their impairment. Therefore, improvement of sensory function might improve motor performance. In this study, we propose that an augmented vibrotactile biofeedback may improve motor learning in children with acquired dystonia, but not in children with genetic dystonia, who do not have associated sensory deficits.

**Design and participants:**

To test this hypothesis, we recorded muscle activity and kinematic recordings and computed outcome measures that represent motor skills, during practice of a point-to-point movement and trajectory-following task. We examined the effects of applying vibrotactile biofeedback on dystonic muscles, in healthy children and those with genetic and acquired dystonia.

**Results:**

The device significantly improved motor learning in children with acquired dystonia, in the cyclic task only, as evidenced by reduced error and improved task correlation index (*p < 0.01*), while no significant effects were observed in other groups.

**Conclusion:**

Our results show that the vibrotactile device can become an effective method of motor improvement only for cyclic and smooth tasks but not for point-to-point tasks in children with acquired dystonia.

## Introduction

Sensory awareness has been shown to be an effective way to improve motor performance (1–4). Previous studies highlight the need to explore artificial sensory feedback as a non-invasive method for improving skill learning (5). On the other hand, understanding how brain network disorders affect motor learning first requires examining the role of sensory deficits. Although artificial sensory augmentation benefits some adults in rehabilitation (5, 6), its effectiveness in children with dystonia remains unknown.

Dystonia is a movement disorder that can be genetic or acquired (due to another underlying disorder such as Cerebral Palsy) (7–9). While medication and surgery can be effective at ameliorating motor symptoms, non-invasive treatments remain valuable. Physical therapy is often ineffective at improving motor function in children with dystonia, possibly due to “failure of motor learning” theory, which holds that without sufficient sensory feedback during movement, practice will fail to improve performance despite adequate repetition (10, 11).

Sensory deficits appear in multiple dystonia forms, including adult-onset focal hand dystonia and dyskinetic CP in children (5), but not in genetic dystonia. Therefore, we propose that some children with dystonia fail to improve with practice due to their associated sensory deficits. If so, then biofeedback may enhance the sensory perception of movement and improve motor learning in children with dyskinetic CP, who exhibit sensory deficits (5). Conversely, without sensory deficits, biofeedback likely has little effect. We examine whether an EMG-activated vibrotactile biofeedback (BF) device improves symptoms in children with acquired dystonia (7), by measuring their motor performance and compare them with a group of healthy children and a group with genetic dystonia.

The speed-accuracy trade-off of Fitts’ law explains the relationship between the speed and the endpoint accuracy during a “fast” movement to a target (12, 13). One of the explanations for this phenomenon is that the trade-off represents compensation for signal-dependent noise, so moving slowly reduces noise and therefore, increases accuracy (12–16). Earlier studies have shown that speed-accuracy trade-off can be modified by practice and the quantitative relationship between speed and accuracy may be an indicator of skill in some tasks (16–20). Moreover, in earlier work, we showed that children with acquired dystonia are aware of their limitations and adjust their movements based on the large signal-dependent noise in their movement (15, 17, 21). Because of the increased motor variability in children with dystonia, Fitts’ law is a good measure of the effect of dystonia on performance (16, 17, 19, 20, 22–24). This is particularly relevant to children with acquired dystonia, because improvement in the speed-accuracy relationship could represent a reduction in a deficit associated with dystonia.

Using Fitt’s law (12), we investigated whether augmented sensory feedback on a dystonic muscle helps children with acquired dystonia in performing two different tasks. We asked whether biofeedback could boost maximum speed for a given accuracy or improve accuracy at a given speed. We, then, evaluated performance using kinematic and muscle recruitment measures to identify any significant benefit from vibrotactile biofeedback.

## Methods

### Study Design and Participants:

This is a multi-center crossover study comprised of 2 weeks of training with a wash-out period of 1 to 4 weeks. The weekly training was performed with or without the use of an EMG-based vibrotactile device. Three different clinical centers were involved in the studies: Neurological Institute IRCCS C. Besta, Milano, Italy; IRCCS Eugenio Medea, Bosisio Parini, Lecco, Italy; and Children’s Hospital, Los Angeles (CHLA), California, USA. The Ethical Committees of each center individually approved the protocol of the study (Neurological Institute IRCCS C. Besta: reference number 24 approved on 16–12-2015; IRCCS Eugenio Medea: reference number 054/14-CE approved on 01–04-2015; Children’s Hospital LA: reference number: CCI-11–00002 approved on 06–08-2011).

Three different groups of subjects were recruited (56 total): 17 children and young adults with acquired dystonia due to cerebral palsy, who were recruited and trained at IRCCS Eugenio Medea or at Children’s Hospital LA and University of Southern California (USC); 11 children and young adults diagnosed with genetic dystonia, who were recruited at Neurological Institute IRCCS C. Besta and trained at Politecnico di Milano; and 28 healthy children and young adults, who were recruited and trained at Children’s Hospital LA, USC, or at Politecnico di Milano. Inclusion criteria required participants to be in developmental age (6–20 years old), have no cognitive impairment preventing comprehension, maintain stable drug therapy for dystonia without botulinum toxin in the dominant arm up to six months prior to enrollment, and be diagnosed by a pediatric movement disorder specialist using standard criteria (25). They were eligible if they could perform the experimental tasks with at least one upper limb. All patients or their legal guardians provided signed informed consent for Health Insurance Portability and Accountability Act (HIPAA) authorization for the research use of protected health information and all the recorded data, in accordance with the Declaration of Helsinki, if they were recruited in CHLA. All the patients or parents of participants (legal guardians) recruited at IRCCS Eugeneo Medea and IRCSS C. Besta signed a written informed consent for the research and medical use of the recorded data and protected health information in accordance with the Declaration of Helsinki.

**Figure 1:**
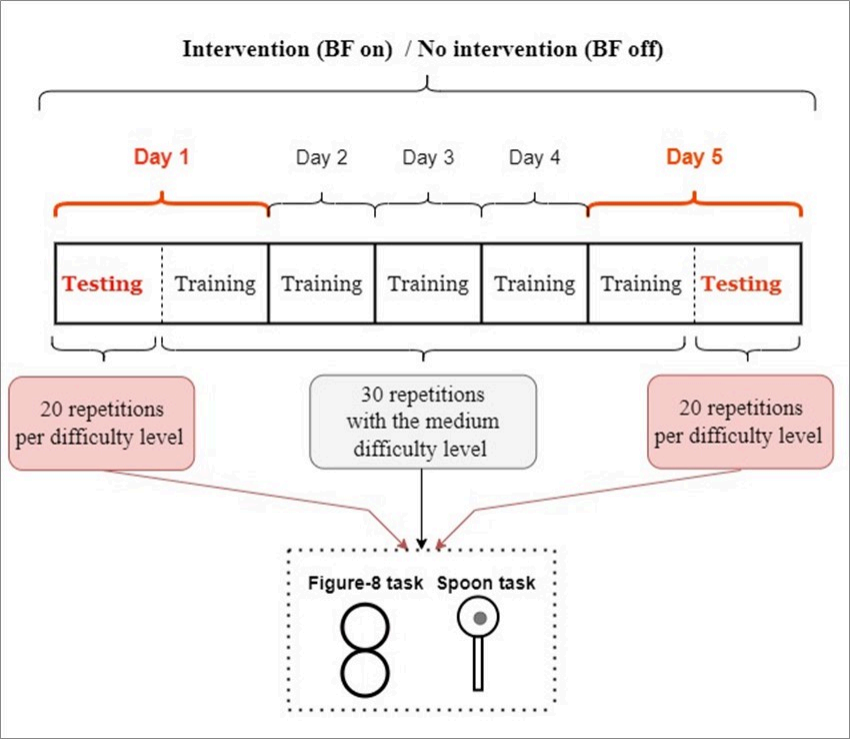
Experiment protocol sequences for week with or without intervention (biofeedback device). On each day they performed both spoon and figure-8 task. On the training days, they only practiced with the medium task difficulty (medium sized spoon and the middle metronome speed). On the testing days, they were tested with two additional easier and harder task difficulties. Each center’s Ethical Committee approved the study protocol.

This study included two weeks of training with a one- to four-week wash-out period, with or without an EMG-based vibrotactile device (23, 24). Subjects performed test and training blocks, on five consecutive days with or without the vibrotactile device. After four weeks, they returned and performed the same tasks for another five consecutive days. Those who performed the first week with the device performed the second week without the device (23) and vice versa. The choice of the week to use the device was random to account for the learning effect. On each week (block), they performed a testing trial on the beginning of the first day and the end of the fifth day under three different conditions, for each task. They practiced the task after the testing trial on the first day, on days two, three, and four, and before the testing trial on day five. The detailed protocol and preliminary data are published in (23). See Fig. 1 for a schematic of the experiment protocol.

### Data Recording:

To evaluate the performance on testing days, kinematics of the upper arm and surface electromyography (EMG) from 8 upper limb muscles (e.g. flexor carpi radialis (FCR), extensor carpi ulnaris (ECU), biceps, triceps, supraspinatus, anterior, posterior, and lateral deltoids) were simultaneously recorded.

Kinematic data were collected using four Vicon Nexus 1.8.5 motion capture cameras (©Vicon Motion Systems Ltd, UK) with sampling frequency of 100 Hz at USC, eight optoelectronic cameras by BTS Bioengineering with sampling frequency of 60 Hz at IRCSS Medea, and POLARIS VICRA with sampling frequency of 20 Hz at Politecnico di Milano. Passive reflective markers were placed on the upper extremity joints.

EMG signals were collected using Biometrics 8-channel wireless EMG system, sampled at 1000 Hz at USC, BTS Free EMG sampled at 1000 Hz at IRCCS Medea, and Porti 32 TMSi sampled at 2048 Hz at Politecnico di Milano. Maximum voluntary contraction (MVC) for all 8 muscles, every day, prior to the onset of experiments was recorded. To record the MVC for each individual muscle, we positioned the subject’s arm in a way that facilitates the isolation of that muscle. Initially, we recorded the baseline EMG, followed by instructing the participants to contract the isolated and stabilized muscle against resistance, maintaining maximal isometric contraction for 5 seconds.

**Figure 2:**
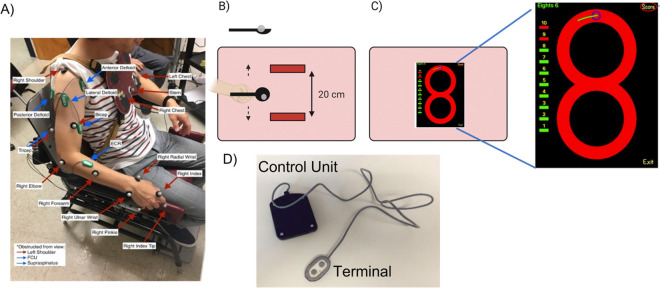
A. Kinematic reflective markers and EMG sensors placement used at USC. Total of 12 reflective markers were attached to the joints and limb to record the kinematic data. One additional marker was attached to the index finger in the figure 8 task to capture the finger trajectory. For the spoon task, one additional marker was attached to the spoon and one sphere marker was used as the marble in the spoon (total of 14); B. Spoon task setup: a board with two plastic blocks attached to it was placed on the table. The distance between blocks is 20 cm along the vertical axis. The participants started the task at the upper target while fully extending their arm; C. Figure-8 task setup and the iPad application: The scores are shown on the left side of the iPad app GUI. The subjects started the task at the blue dot above the figure-8, while fully extending their arm, and followed the red figure-8 trajectory line. They had to adjust their speed in a way to be on the top blue dot at each beep of the metronome; D. The vibrotactile biofeedback device; the terminal head contains the filtering circuit and the vibrating motor which was to the target muscle. The control unit consists of the microcontroller and the circuit for battery recharging (23).

The EMGs and motion capture system were synchronized by a trigger at the start of the movement. More accurate synchronization was done with signal processing and cross correlation of the signals. The EMG and motion capture sensors setup and placement are shown in Fig. 2A.

### Experiments

#### Spoon task

This is a point-to-point, trajectory-constrained task, during which participants carried a reflective marble on a spoon between two targets without dropping it, which enforced a smooth velocity profile (16, 17). The subjects were seated upright on an adjustable chair and the severely affected dystonic subjects used their own wheelchair. A board with two targets was placed in front of participants, positioned so they had to fully extend their arm to reach the farther target. Each target was bounded by plastic blocks spaced 20 cm apart along the movement axis, to limit forward and lateral acceleration. A reflective marker was attached to the spoon, and a separate spherical reflective marker served as the “marble”. Participants were asked to move the marble back and forth between the two targets as quickly as possible without dropping it. Figure 2B illustrates the setup (16, 17, 26). Nine spoons with various sizes were used and the index of difficulty (IoD) for each trial was calculated based on the spoon dimensions (12, 16, 17), as the ratio of the spoon diameter to its depth. Therefore, the IoD is greater for shallower spoons with smaller diameters, as stabilization of marble in the spoon without dropping requires more precise attention to smoothness of the trajectory. The details of the setup, spoon sizes, and IoDs are reported in our previous work (16, 17).

Prior to the experiment for each subject, performance was tested on a range of different spoon difficulties. Easy, medium, and difficult spoons were chosen for each subject. The difficult spoon was chosen as the largest IoD for which the subject could successfully transport the marble dropping it on fewer than 30% of trials. The medium and easy spoons were the next 1 and 2 spoon difficulties below. Testing was performed with all three spoon sizes. Training took place only with the medium spoon size for each participant (on days one through 5). No specific cost was associated with dropping the marble, however if they dropped the marble three times or more out of ten repetitions, they would be asked to redo the trial (16, 17). Additionally, we asked the subjects to perform the task without the marble which allowed us to measure the maximum unconstrained speed and represents the true Fitt’s law, based only on accuracy at the target endpoint without a constraint during the experiment, to assess the ceiling effect during the practice.

#### Figure-8 task

This is a cyclic task constrained by movement speed (controlled by metronome), requiring participants to follow a figure-8 trajectory with their index finger (22, 26). An iPad was placed in front of the participant and they were instructed to follow a figure-8 trajectory (consisted of two circles of 20 cm diameter and 1 cm width) with their index finger, as depicted in Fig. 2C. The iPad’s position was adjusted so that the subjects had to extend their arm fully to reach the top point of figure-8 with their index finger, maintaining the contact between the finger and screen. Participants had to follow the trajectory clockwise or counterclockwise depending on the arm used. An additional marker for tip of their index finger were used to capture the finger movement trajectory (22).

Prior to the experiment, performance was tested on a range of different speeds (10 to 45 bmp), controlled by a metronome. The fastest tempo was chosen as the most difficult task for which the subject could successfully follow the figure-8 with fewer than 30% failure of following the trajectory line. The medium and easy levels were the next lower 5 and 10 bpm. Testing was performed on all three speeds, but training was performed only with the medium speed. On each trial, participants were asked to perform the figure-8 for 10 repetitions. Participants were encouraged to make continuous movements, not stopping at any point on the iPad. Therefore, the first 5 repetitions were done with the metronome on. For the next 5 repetitions we turned off the metronome and they had to remember the speed of movement and continue the task, allowing for smoother and continuous movements. The figure-8 application on the iPad could detect if participants were moving out of the allowed borders and would mark that repetition as a failed one.

#### Vibrotactile Biofeedback Device

The vibrotactile biofeedback device, shown in Fig. 2D, contains a vibration motor and active differential electrode head that records target muscle activity (23). The vibration motor is at the head of the device apply feedback directly at the site of muscle-electrode contact for a clear and relevant stimulus. This electrode head is connected to a control unit that computes the amplitude of the recorded EMG signal through Bayesian estimation and controls the silent vibration motor with a rotation speed and amplitude proportional to the magnitude of the EMG. The processor and nonlinear filter in the device are designed to enable proportional biofeedback (23, 27). Each participant’s most dystonic muscle received vibrotactile feedback, as shown in Table 1.

### Data Processing

**Figure 3:**
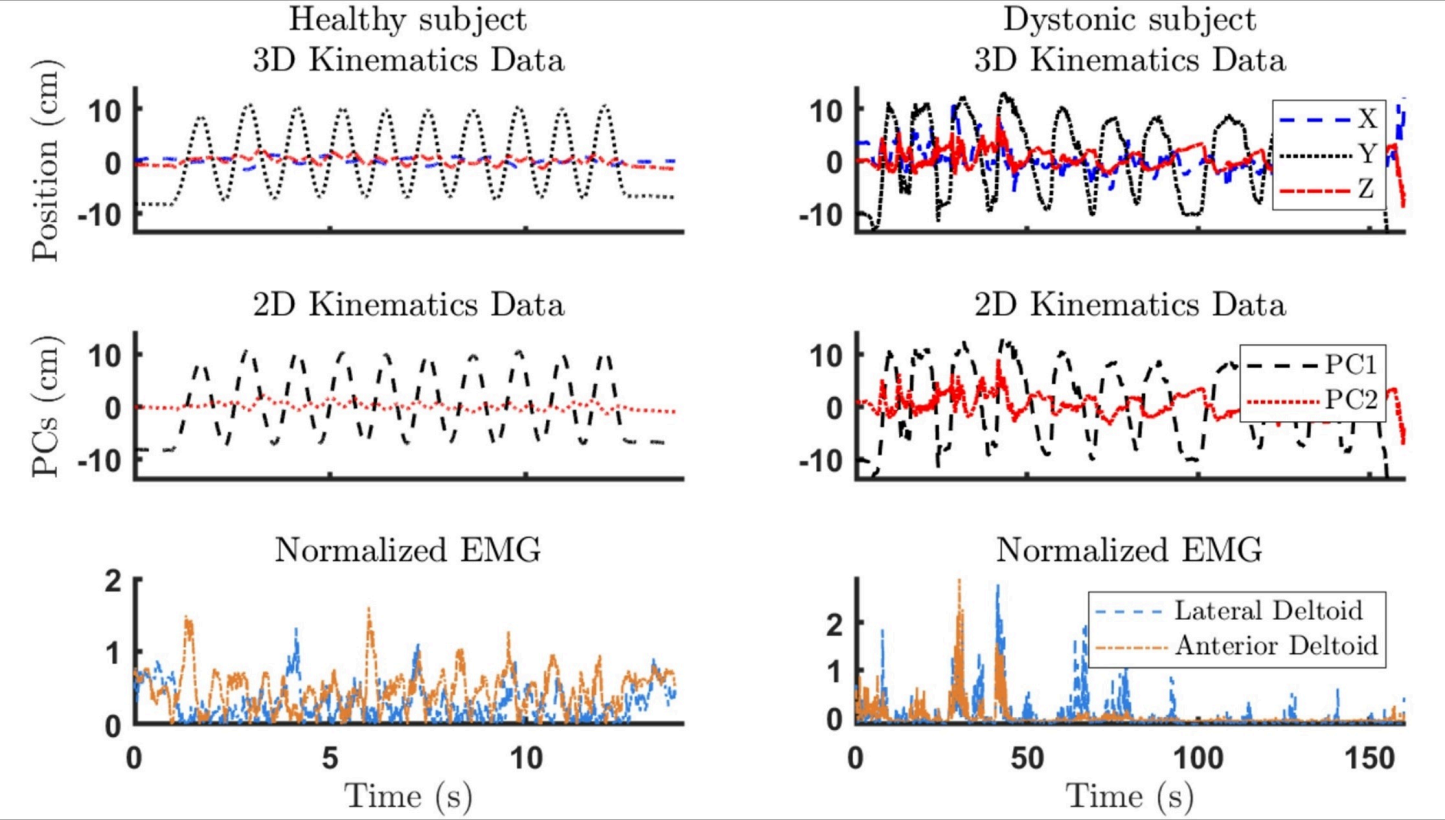
Samples of kinematic data and EMGs for a full trial of spoon task for a healthy (left) and a dystonic subject (right); (top) 3D kinematics data recorded during performance of the spoon task, in X, Y, and Z directions. (middle) First two principal components of X, Y, Z recordings. These two PCs were used throughout the analysis. (bottom) Corresponding EMG recordings of anterior and lateral deltoid.

Data processing was conducted in MATLAB R2020a (MathWorks, Natick, MA, USA). All EMG signals were band-pass filtered at 2–200 Hz, then normalized to MVC. Kinematic data were reconstructed and interpolated as needed in Cortex 5.5 (Motion Analysis Corp., Rohnert Park, CA, USA). After applying a 5 Hz low-pass filter to the kinematic data, we used principal component analysis (PCA) to extract 2D joint coordinates from the 3D data. Kinematic data and EMG were synchronized by cross-correlation, resampled to 1000 Hz, and split into figure-8 or spoon-task repetitions. The segments in which participants dropped the marble or lifted their finger from the iPad were treated as outliers. Figure 3 shows samples of preprocessed EMG and kinematic data from a healthy subject and a dystonic subject performing the spoon task. Finally, we calculated the average movement time in each repetition, then derived outcome measures for statistical analysis.

#### Spoon Task Outcome Measures:

Index of Performance (IP): We fitted a regression line to the movement time (MT) and the IoD and calculated the index of performance (IP) as the inverse of the MT-IoD slope (17). For each subject, we compared day 1 and day 5 IPs to see if improvement was greater during the “biofeedback week (BF on)” than during the “no-biofeedback week (BF off) (12–14).

Co-contraction index (CCI): Co-contraction index is a useful parameter to evaluate the performance, especially in point-to-point tasks. Muscles co-activation plays an important role performing these tasks as sudden change of direction requires accurate and in-time activation of agonist-antagonist muscles (28, 29). This measure for a pair of agonist-antagonist at each repetition is computed as (30):

(1) $$CCI=mean\left(\frac{min\left(EMG_{i},\;EMG_{j}\right)}{max\left(EMG_{i},\;EMG_{j}\right)}\right)\;*\;\left(min\left(EMG_{i},EMG_{j}\right)+max\left(EMG_{i},EMG_{j}\right)\right)$$

in which, CCI lis the co-contraction index, “min” is the minimum and “max” is the maximum of an EMG pair ($EMG_{j}$ and $EMG_{j}$) at an instant in time.

#### Figure-8 Task Outcome Measures:

Time * Error: This measure, derived from Schmidt’s law, a variation of Fitt’s law, captures performance in open-loop motions within thin lines (31). We can assume that the movement is cyclic, therefore, time in this formula is the time required to complete a single figure-8 repetition. In this experiment the error is computed as (26, 31):

(2) $$\mathrm{Error}=\sqrt{\frac{1}{N}}\;*\;\sum_{t=1}^{N}d(t{)}^{2}$$

Here, N is the number of samples, and d(t) is given by:

(3) $$d(t)=\left(\frac{min\;\left(d_{U}\;-\;d_{L}\right)\;-\;\mathrm{radius}}{\mathrm{radius}}\right)$$

in which dU and dL are the distances between the position and the upper and lower circles, respectively. The smaller the time*error, the better the performance. Therefore, it should decrease with skill learning (31). We compared this measure in figure-8 task with index of performance (IP) in the spoon task for the final evaluations.

Task correlation index (TCI): The figure-8 task captures both task-relevant and task-irrelevant frequencies in the kinematic and EMG signals. Because the trajectory is symmetrical, an ideal performance would show the X frequency as double the Y frequency, corresponding to four x-direction crossings for every two y-direction crossings. This design separates task-irrelevant (possibly dystonic) frequencies from task-relevant ones. The index is computed as:

(4) $$TCI_{i}=\left(\frac{PSD_{EMGi}\;|\;f_{x}\;+\;PSD_{EMGi}\;|\;f_{y}}{PSD_{EMGi}}\right)$$

Here $PSD_{EMGi}$ is the total power of the muscle i’s EMG, $PSD_{EMGi}|f_{x}$ and $PSD_{EMGi}|f_{y}$ are the peak powers in X and Y directions (at the task frequencies). This index ranges from 0 to 1, with higher values indicating better performance (22).

**Figure 4:**
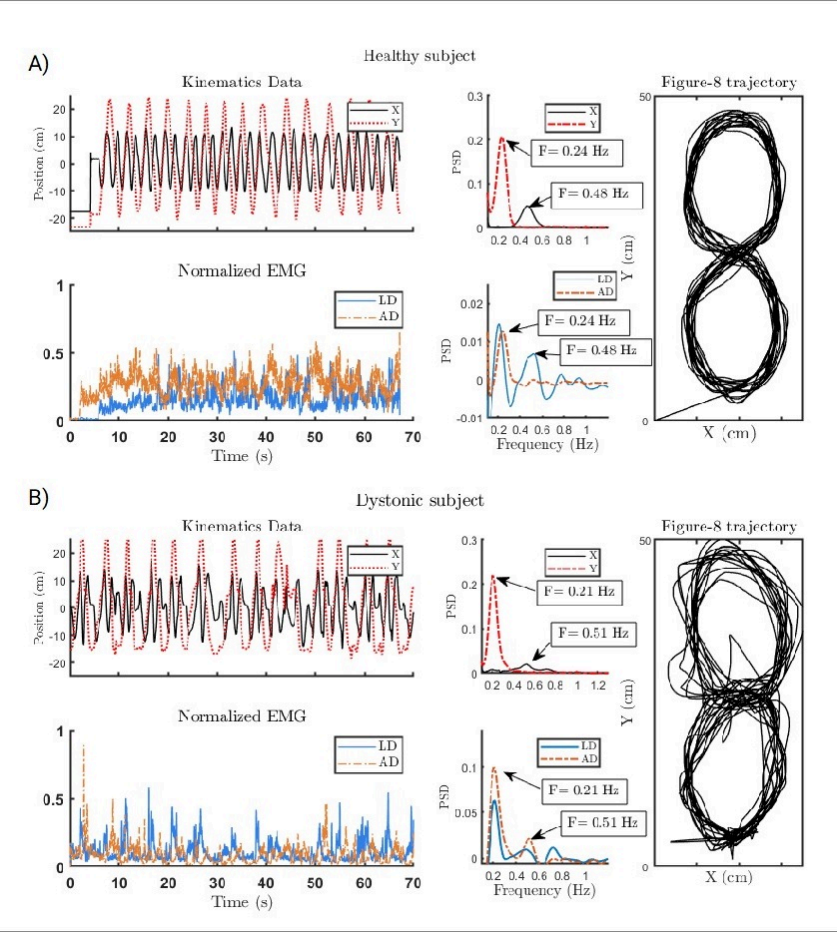
Recorded data during performance of the figure-8 task for (A) a healthy and (B) a dystonic subject. For each subject (top) 2D kinematics data in X and Y directions and their normalized spectrum, (bottom) the corresponding EMG recordings of anterior and lateral deltoid and their spectrum, and (right) their corresponding movement trajectory are depicted. Spectra of X and Y for the healthy subject show peaks at 0.48 and 0.24 Hz, respectively; one is exactly twice the other one. The Spectrum of the AD EMG only peaks at 0.24 Hz contributing the movement only in X direction, while the LD EMG spectrum peaks at both task frequencies, contributing to movement in both X and Y directions. For the dystonic subject, the PSD of the LD and AD show two peaks only 0.51 and 0.21 Hz, contributing to the movements in both directions. Some overflow of activity in the LD is also observable at around 0.7 Hz.

In Fig. 4, we present sample kinematic data, EMG spectra, and figure-8 trajectories from a healthy subject and a subject with acquired dystonia. While the dystonic subject’s X and Y spectra show distinct peaks, they are less sharp than in healthy controls, and their frequencies are not precisely double, indicating movement overflow.

### Statistical Analysis:

The statistical analyses on all the outcome measures were performed using lme4 (32) and emmeans (33) packages in R-studio (R core team, 2021). A Linear mixed effect model with repeated measures was employed to test the effect of practice on motor learning, with and without the vibrotactile biofeedback, for all outcome measures. For the spoon task outcome measures, we assessed the effect of biofeedback device (on versus off), group (i.e., genetic, acquired or healthy), testing day (i.e., day 1 or day 5), IoD, and their interactions, as independent variables (fixed effects), on all the outcome measures. In our model, random effects are intercepts for subjects and by-subject random slopes for the effect of index of difficulty (Eq. 5). We used Kenward-Roger’s F-test for pairwise comparisons, comparing estimated marginal means of performance indices to assess the effect of biofeedback.

(5) Outcomemeasure~Testingday*Group*Week*IoD+(IoD|Subject)

All figure-8 task measures were normalized for movement speed to remove the effect of task difficulty. We then built a linear mixed-effects model with the same fixed and random effects used in the spoon task (Eq. 6) and performed ANOVA and pairwise comparisons to assess significance of each independent variable.

(6) Outcomemeasure~Testingday*Group*Week+(1|Subject)

## Results

Fifty-six participants were recruited: 28 healthy controls, 17 with acquired dystonia due to cerebral palsy, and 11 with genetic dystonia. Table 1 presents their characteristics and corresponding experimental details.

### Spoon task

**Figure 5:**
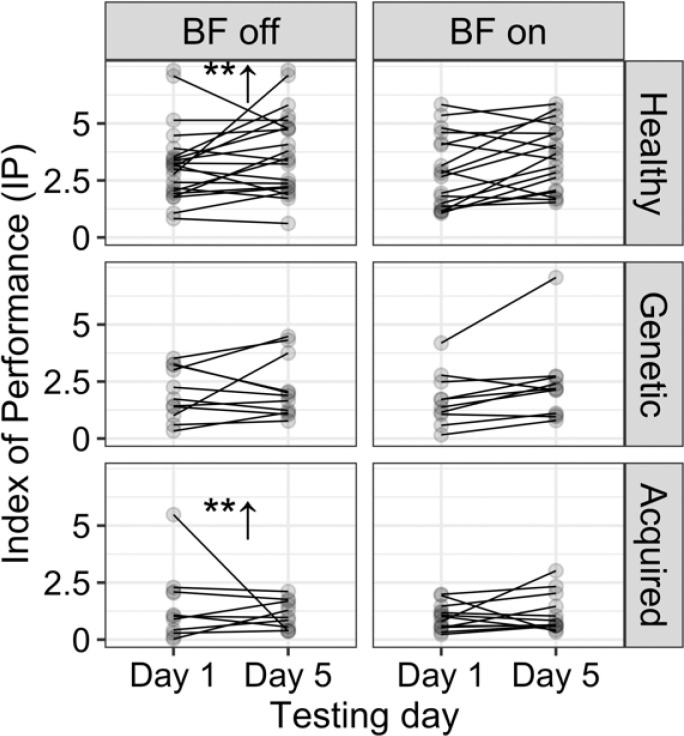
The index of performance for the spoon task shows no significant changes or improvement with the biofeedback device. However, learning occurred on the week without the device in children with acquired dystonia and healthy subjects (p < 0.01).

The regression lines of movement time versus the index of difficulty (R^2^ = 0.96) showed a non-significant change in the IP for all healthy children and children with dystonia whether there was an intervention or no intervention during the practice (17). Although the participants moved faster with practice (indicated by a significant decrease in movement time, demonstrating learning) (reported in (17)), the slopes of the regression lines remained unchanged, which means there was no significant improvement in IP. These results suggest that the children with acquired dystonia did not benefit from the vibrotactile biofeedback in the spoon task experiment, as shown in Fig. 5.

**Figure 6:**
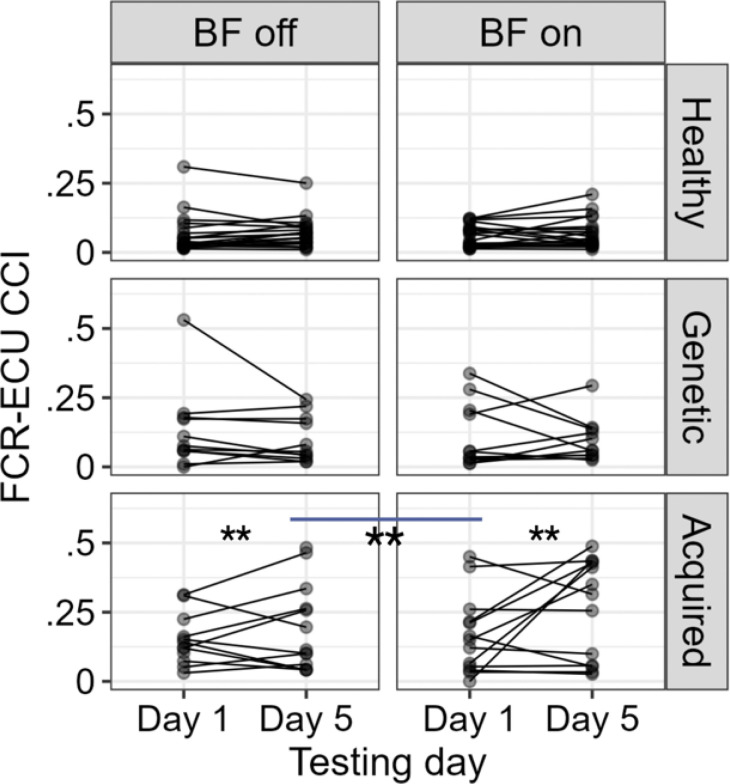
The FCU-ECR co-contraction index was increased significantly due to the practice with the vibrotactile device.

Due to the ineffectiveness of the biofeedback device on the spoon task performance, we then evaluated the change in CCI of FCR-ECU (R^2^ = 0.72), biceps- triceps (R^2^ = 0.64), and anterior-posterior deltoid (R^2^ = 0.72), to evaluate whether the biofeedback device affected the CCIs in an adverse way. The pairwise comparison of the estimated marginal means showed that the device significantly increased the FCR-ECU CCI (*mean BF off vs BF on = 0.06, SE = 0.008, p < 0.01*) only in children with acquired dystonia as shown in Fig. 6. The effect of biofeedback was negligible on the Biceps-Triceps and AD-PD CCIs.

#### Figure-8 task:

**Figure 7:**
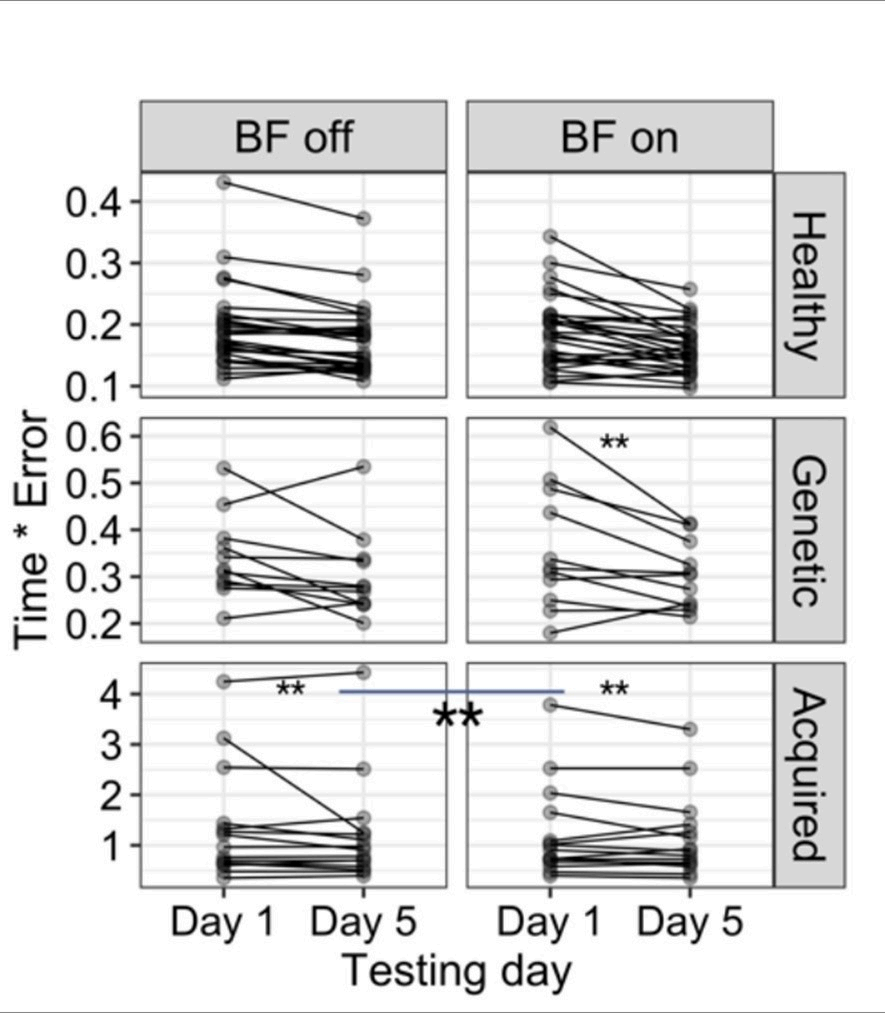
The figure demonstrates that the device improved learning when practicing the figure- 8 task. This is reflected in the significant decrease in time * errorduring the BF-on week compared to the BF-off week in children with acquired dystonia.

**Figure 8:**
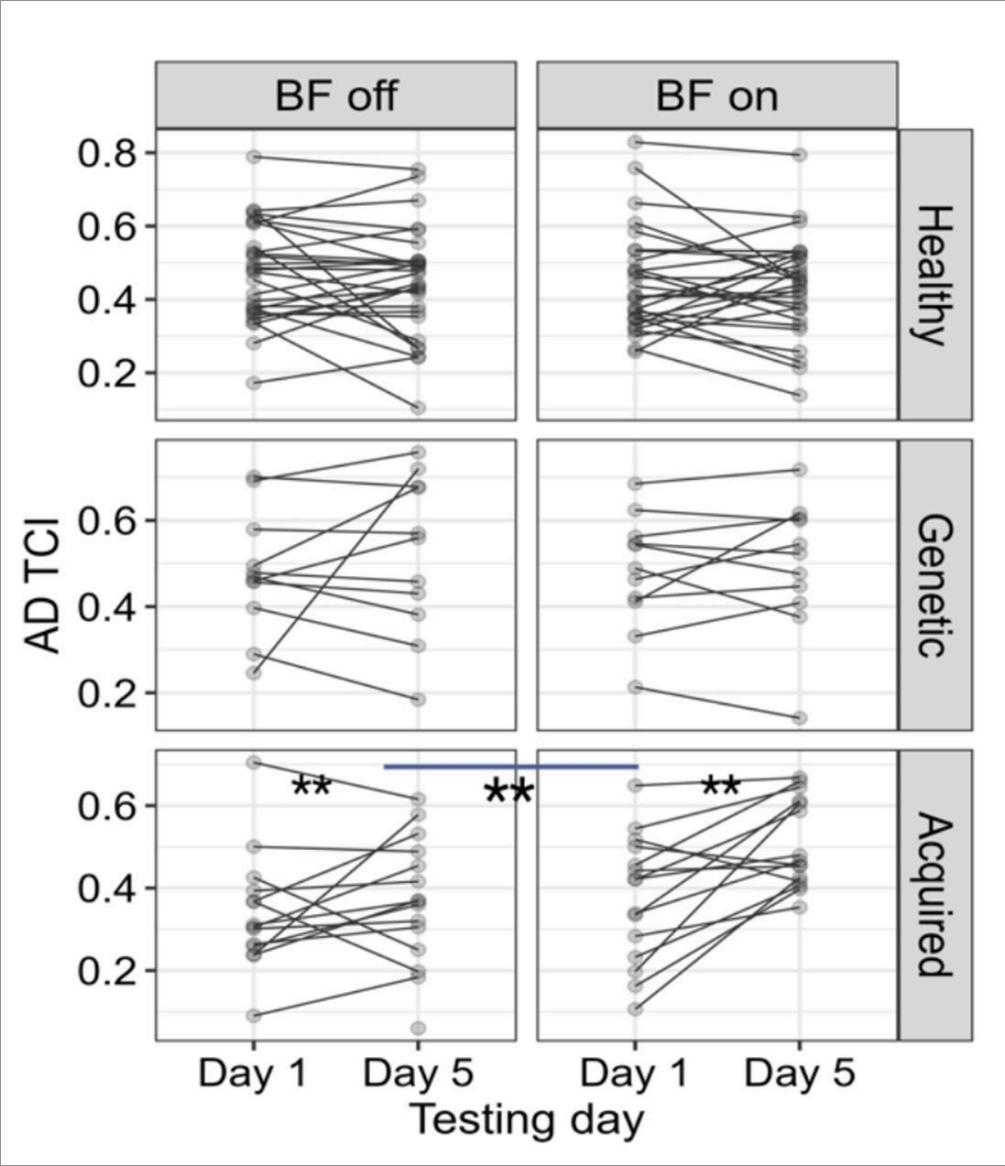
The figure demonstrates that the device improved anterior deltoid task-correlation index (TCI). This is reflected in the significant increase of this index during the BF-on week compared to the BF-off week in children with acquired dystonia.

The linear t (R^2^ = 0.88) and the pairwise comparison for time*error revealed significant decrease (*p < .01*) for the children with acquired dystonia with the biofeedback device on, compared to the week that they practiced without the biofeedback *(mean BF off vs BF on = 0.13, SE = 0.02, p < 0.01*). Two other groups (healthy and genetic dystonia) did not show sensitivity to the biofeedback device (Figure 7). The task correlation index for the anterior deltoid muscle (R^2^ = 0.57) increased significantly (*p < .01)* for the children with acquired dystonia while performing the figure-8 task with the biofeedback device *(mean Day 1 vs Day 5, BF on = 0.11, SE = 0.02; mean BF off vs BF on = 0.05, SE = 0.01)* as shown in Figure 8. However, this measure did not change significantly in other muscles.

## Discussion

In this study, we hypothesized that the vibrotactile device would be an effective device to improve skill learning in children with acquired dystonia because of their sensory deficit. To test our hypothesis, we asked the participants to perform two tasks that are different in nature, following a specific protocol: the spoon task and the figure-8 task. In the spoon task, there is a smoothness constraint (“do not drop the marble”) and subjects are asked to move as fast as possible (26). In the figure-8 task, there is a time constraint indicated by a metronome, and subjects are asked to follow the trajectory as accurately as possible (22). By varying task difficulty, participants could choose their velocity profile in the spoon task (17) and their accuracy in the figure-8 task. If they learned an improved speed-accuracy relationship, represented by the explained outcome measures, both tasks would show improved performance.

Our results suggest that this hypothesis is only partially correct. In other words, our results show that the vibrotactile biofeedback device improved the motor learning in figure-8 task (continuous task), but not the spoon task (point-to-point task), meaning that children with acquired dystonia learned the figure-8 task significantly better, practicing with the biofeedback device. This device seemed to decrease the movement variation in children with acquired dystonia, which could possibly be the reason why we observed increased task correlation index of the anterior deltoid (task relevant muscle) in this group. On the other hand, this device does not seem to be very effective in improvement of learning the spoon task. Nonetheless, it was observed that learning took place in the week without any intervention (BF off), as reported in our previous study (17). These results could be explained by the different nature of the two experimental tasks. The figure-8 task is a cyclic and continuous task which does not require very accurate muscle control and co-contraction, while the spoon experiment is a point-to-point task which requires more accurate muscle control, timing, and controlled agonist-antagonist co-contraction in order to change the direction of movement at each end of a forward or backward movements. Previous studies have shown that children with dystonia have failure in the timing of co-contraction, which is due to the involuntary activation of an antagonist muscle during the movement (34, 35), leading to in- creased co-contraction during the movement. Therefore, we further hypothesize that in order for the treatment to be effective in the spoon task, the muscle co-contractions pattern have to change, and this change may be inconsistent among the subjects, as each has a different baseline, adopt a different approach, and their dystonia manifests onto different muscles. Another possibility is that the marble is effectively a biofeedback signal for movement, and this may have been more important in performance (drawing the attention to perform the task without dropping the marble) than the muscle EMG biofeedback, effectively drowning out any possible effect.

In this study, biofeedback enhanced performance in the figure-8 task but exhibited no improvement in the spoon task, indicating that mere practice is not adequate for achieving peak performance. The results in genetic dystonia showed lack of improvement in either task. While this could occur due to multiple mechanisms, it is consistent with the hypothesis that biofeedback will improve movement only when a sensory deficit is at least partly responsible for poor movement and skill learning. This supports our initial hypothesis that acquired dystonia deficits might be partially due to learning failures stemming from sensory deficits. This proposition raises the potential for noninvasive treatments for acquired dystonia that center on augmenting sensation.

## Data Availability

The data and codes are available upon request.
